# Parental practices, preferences, skills and attitudes on food consumption of pre-school children: Results from Nutriscience Project

**DOI:** 10.1371/journal.pone.0251620

**Published:** 2021-05-25

**Authors:** Carla Almeida, José Azevedo, Maria João Gregório, Renata Barros, Milton Severo, Patrícia Padrão

**Affiliations:** 1 Faculdade de Ciências da Nutrição e Alimentação da Universidade do Porto, Porto, Portugal; 2 EPIUnit—Instituto de Saúde Pública, Universidade do Porto, Porto, Portugal | ITR—Laboratório para a Investigação Integrativa e Translacional em Saúde Populacional; 3 Faculdade de Letras da Universidade do Porto, Porto, Portugal; 4 Departamento de Ciências da Saúde Pública e Forenses e Educação Médica, Faculdade de Medicina da Universidade do Porto, Porto, Portugal; Centre Hospitalier Regional Universitaire de Tours, FRANCE

## Abstract

The association between family environment and child’s eating behaviors is well established but a multidimensional approach to study this relation is lacking. This study aimed to assess the proprieties of a questionnaire created to evaluate parental practices, preferences, skills and attitudes regarding fruit and vegetables (F&V), sugar and salt. Participants (n = 714) were families of pre-school children (aged 2–6 years old) of the Nutriscience Project–a web-based gamification program–who answered a questionnaire assessing socio-demographic characteristics, nutrition knowledge, and a scale evaluating parental practices, preferences, skills and attitudes, at the baseline of the project. Exploratory factorial analysis was applied to the scale: 21 items and 5 factors were extracted (52.4% of explained variance) with a Kaiser-Meyer-Olkin (KMO) value of 0.770: 1. Modelling/active promotion of F&V consumption (α = 0.73), 2. Skills for choosing/preparing healthy food (α = 0.75), 3. Food preferences and satiety perception (α = 0.70), 4. Awareness regarding sugar/salt intake (α = 0.61), 5. Allowance regarding F&V consumption (α = 0.55). Kruskal-Wallis and Mann-Whitney tests were conducted to compare factors according to socio-demographic characteristics. Higher scores for parental modelling and active promotion of F&V consumption were observed in older parents, those with higher nutrition knowledge and who reported to live without income difficulties. Regarding food preferences, higher scores were observed in mothers, with higher nutrition knowledge and from higher educated groups. Higher awareness regarding salt and sugar consumption were observed in older parents, with higher education, higher nutrition knowledge and with female children. Older parents and with female children also registered higher scores of skills for choosing/preparing healthy food. The scale showed satisfactory proprieties and may contribute to assess family food environment using a multidimensional approach. It also highlighted the importance of considering socio-demographic characteristics in interventions to promote healthy eating.

## Introduction

An unhealthy diet is considered one of the most important lifestyle risk factors for Noncommunicable Diseases (NCD), which are the main cause of mortality worldwide [1]. The World Health Organization (WHO) considers that reducing salt and sugar intake and increasing fruit and vegetables (F&V) consumption must be priority areas for the promotion of a healthier diet. Several authors revealed that children and adolescents do not meet WHO recommendations for sugar, salt and F&V intake. According to Crowe, O’Sullivan [2], in Ireland, 75% of children aged 3-years old do not meet WHO recommendations for free sugars (<5% of total energy value) and according to Marinho, Severo [3], the lower adherence of free sugars recommendations were in children (51.6%) and adolescents (51.3%). A recent National Food Survey in Portugal, showed that 72% of children and 78% of adolescents do not meet the recommendations of F&V ≥400g/day [4]. According to Gonçalves, Abreu [5], 83% of a Portuguese sample of adolescents had a sodium intake above the upper limit recommended by WHO (2000 mg/day).

Parents have the main responsibility for their child’s eating behaviors [6] which should be early modulated [7, 8], in order to reverse the diet-related NCD growing trend.

Literature suggests that there are associations between family’s food environment and children’s energy-balance related behaviors [9–11]. Several studies showed a consistent positive association between parents’ and child consumption, regarding F&V [12–15], sweet beverages [16] and snacks with high energy value [17]. This association could also be related to home food availability [18–20]. Choosing which foods to buy, is very complex and a decision involves a lot of processes and dimensions (emotional, rational, social, e.g.). Identified this paradigm, this issue has been widely studied from different perspectives due to its complexity. There are studies focusing on socio-demographic characteristics [21, 22], personality/intrinsic characteristics [23, 24], and individual health beliefs, knowledge and skills [25–29]. The importance of consumer’s beliefs and perception is emphasized by the example of frozen versus fresh vegetables. Haynes-Maslow, Parsons [30] demonstrates that, despite the convenience, longer expiration date and similar nutritional value, consumers tend to choose fresh vegetables instead of frozen vegetables. This seems to be due to an intrinsic preference for natural, instead of transformed foods [31]. Besides preferences, Vidgen and Gallegos [25] describes how knowledge and skills interacts with attitudes and behaviors by introducing the concept of nutrition literacy. Nutrition literacy have been described as a powerful allied for healthier eating habits promotion and reveals the importance of having the know-how to appropriately plan and manage meals, select foods, preparate meals and eat.

Considering the diversity of factors described as determinants of eating habits, there are different instruments assessing parental and family’s practices/behaviors related to child’s food consumption using different approaches [32–38]. The diversity of dimensions and determinants of healthy eating highlights the need for a comprehensive and multilevel approach. However, a comprehensive and complete evaluation typically implies higher complexity and extent of the instrument. This may translate into a disadvantage. This study aimed to develop a short and easily interpretable instrument named “The multidimensional parental scale to assess determinants regarding F&V, sugar and salt“, focusing on the main topics for healthy eating promotion–F&V, sugar and salt as recommended, combining a broad range of parental dimensions: practices, skills and attitudes (framed in the concept of nutrition literacy) and food preferences considering: 1. their recognized value as a powerful predictor of food choice and eating habits and 2. the fact that they could be modulated during lifetime. Items focused mainly on parental food preferences, awareness regarding child’s sugar and salt intake, parental ability to promote F&V consumption, to select low-sugar and low-salt products, to prepare meals with F&V and low salt content, and to manage meals were evaluated in the questionnaire.

The objectives of this study were to 1. characterize the main proprieties of a new tool developed to assess parental practices, preferences, skills and attitudes regarding F&V, sugar and salt and 2. to characterize them according to socio-demographic characteristics and nutrition knowledge of pre-school children’s parents.

## Material and methods

The Nutriscience Project: Play, Cook and Learn was a web-based gamification intervention, aimed to evaluate and improve parental nutrition literacy (knowledge, practices, skills and attitudes regarding F&V, sugar and salt) and food preferences of children’s aged 2–6 years old belonging to a national network of kindergartens, *Santas Casas da Misericórdia* of Portugal.

The intervention model of Nutriscience project consisted of a web-based gamification platform developed as a social network environment where schools and families signed up for membership [39]. In this study only baseline data was used for analysis.

### Sample and recruitment

A convenience non-random sample of 37 kindergartens were invited to participate in the program, from those 32 were allocated to intervention group and 5 to the control group, targeting a minimum of 1 kindergarten from each administrative region of Portugal. All the families of pre-school children were invited to participate in this project through the participating kindergartens. Explanations on how to participate and access to the platform were provided in three regional meetings with educators and school directors. In addition, an introductory video and a power point presentation was given to educators to present at parents’ meetings and a pamphlet was distributed to all the eligible parents through the kindergartens.

The project was approved by the Ethics Committee of University of Porto and by the National Commission for Data Protection (CNPD). Informed consent indicating agreement of the parents to participate in this study was also requested before inclusion.

### Instrument development for parental practices, preferences, skills and attitudes evaluation

To assess parents’ practices, preferences, skills and attitudes on children’s food consumption, a parental self-reported questionnaire was applied, which was filled out on the Nutriscience online platform. For the control group, a paper-based version of the questionnaire was handed out and returned through the kindergartens. The questionnaires were administered before and after the intervention (3 month period), assessing 1) socio-demographic characteristics (respondent´s age and degree of kinship to the child; number of household members; household classification (monoparental, couple with children or households with other members); number of household children; sex and age of participant child; education level, job situation and type of work’s institution of the respondent; income perception; food insecurity level, considering as a situation where difficulties related to access to food due to a socioeconomic factor were reported [40]), 2) nutrition knowledge [39], and 3) parental practices, preferences, skills and attitudes regarding F&V, sugar and salt. A pre-test was conducted in a sample of 10 families with 2–6 years-old children, in order to evaluate the time taken to fill up the questionnaire and the common understanding and interpretation of the questions. Based on the results obtained in the pre-test, minor changes were made to improve the clarity of the questionnaire.

For nutrition knowledge assessment, an adapted version of Nutrition Literacy Assessment Instrument (NLAI), published by the Centers of Disease Control and Prevention was used [41]. The adaption of the NLAI was performed regarding to the following aspects: 1) Translation and back-translation; 2) Portuguese food habits; 3) Portuguese food guide (Food Wheel); 4) The 3 major topics of the project namely a) promoting the consumption of F&V; b) reducing the intake of sugar and c) salt. A total of 20 multiple response questions covering 4 different dimensions: Nutrients (6 items), Food Portions (3 items), Portuguese Food Wheel Guide Groups (7 items), and Food Labeling (4 items) were evaluated.

Regarding parents’ practices, preferences, skills and attitudes, a Likert scale from 1 to 5 points was used for each item (1 –Completely disagree to 5 –Completely agree). Participants could also select the option–“Do not want to answer”. Items from questionnaires addressed in European populations (EPODE for the Promotion of Health Equity (EPHE) [42], PRO-GREENS [11] (updated version of Energy parent and child questionnaire) and the EuropeaN Energy balance Research to prevent excessive weight Gain among Youth (ENERGY) questionnaire [10] were translated and adapted. Other items were developed according to the project main food topics and objectives. For items selection, some dimensions were draw *apriori* according to the project’s objectives focusing on parental active encouragement of child’s F&V consumption, parental allowance of child’s F&V consumption, parental self-efficacy to manage child’s intake, parental practices conducting energy-balance related behavior together with the child, parental food preferences and satiety perception, parental skills for choosing/preparing healthy food and parental awareness regarding child’s salt and sugar intake. Items were grouped in dimensions according to Exploratory Factorial Analysis procedures, as described below.

### Statistical analysis

#### Data preparation and participants’ characterization

Statistical analysis was performed using IBM SPSS Statistics v25. This study analysis included only cases who declared that participant children was aged 2–6 years old and whose questionnaires were fulfilled by parents (mother or father). Regarding parents’ practices, preferences, skills and attitudes on child’s food consumption, only cases of individuals who answered all items were considered for the respective analysis (N = 618).

In order to analyze the data, the items “Vegetables do not satiate me”, “Fruit does not satiate me”, “Food without salt has no taste” and “I like the sweet beverages taste” were recoded through the inversion of the Likert Scale (5 –Totally disagree; 1 –Totally agree). Considering nutrition knowledge and food insecurity level scores, individuals who indicated “do not want to answer” were considered missing. Socio-demographic characteristics and nutrition knowledge were compared between participants who answered the questionnaire on paper or online according to Chi-Square test.

#### Exploratory factorial analysis: Instrument characterization

Regarding parents’ practices, preferences, skills and attitudes on children’s food consumption, Exploratory Factorial Analysis was used to reduce information’s complexity by extraction of different factors. The method of Principal Components and the Varimax rotation was used. Exploratory Factorial Analysis procedures were conducted for all items of the scale (n = 22). Items were included if factorial loadings and communalities’ values were >0.3 and >0.5, respectively. Three criteria were used to determine the number of factors to retain:—Eigenvalues higher than one;—Scree plot observation;—interpretability (including items within the same concept). Internal reliability (Cronbach’s alpha coefficient) was calculated and factors were constructed if Cronbach’s alpha coefficient was acceptable (≥0.6) [43]. Thus, both Kaiser-Meyer-Olkin (KMO) and communalities values, total explained variance of the model and interpretability were important determinants for factors’ extraction and to determine which items were included in the model. Particularly, interpretability assumed a crucial importance considering that further analysis and results’ interpretation were based on those factors.

Values of means and standard deviation (SD) of items were used to factors’ description.

#### Characterization of parents’ food practices, preferences, skills and attitudes on child’s food consumption

For studying the factors (extracted by the Exploratory Factorial Analysis) according to socio-demographic characteristics, nutrition knowledge and method of data collection (paper or online), Mann-Whitney and Kruskal-Wallis tests were performed, as applicable. For Kruskal-Wallis’s multiple comparisons, Stepwise-Stepdown comparisons were used, and the Mann-Whitney test was used to describe which groups were significantly different on each factor. A significance value of 0.05 was considered. Means and SD were used to describe the observed differences of factors according to socio-demographic characteristics, nutrition knowledge and method of data collection.

## Results

### Socio-demographic characterization

Table 1 summarizes the socio-demographic characteristics of the participant families. A total of 723 participants fulfilled the baseline questionnaire and a total of 714 families were eligible for this study.

**Table 1:** Socio-demographic characteristics and nutrition knowledge of participants.

	Participants (n = 723)	Paper (n = 224)	Online (n = 499)	Significance value
	*N (%)*	*N (%)*	*N (%)*	
**Respondent**				
Mother	597 (82.6)	204 (91.1)	393 (78.8)	0.001*
Father	117 (16.2)	20 (8.9)	97 (19.4)	
Other	9 (1.3)	0 (0.0)	9 (1.8)	
**Respondent age (years)**^1^^,^^2^				
<30	74 (10.4)	22 (9.8)	52 (10.6)	0.138
30–40	513 (71.9)	153 (68.3)	360 (73.6)	
>40	126 (17.7)	49 (21.9)	77 (15.7)	
**Education level (schooling years)**^1^				
<10	91 (12.7)	34 (15.2)	57 (11.6)	0.389
10–12	240 (33.6)	71 (31.7)	169 (34.5)	
>12	383 (53.6)	119 (53.1)	264 (53.9)	
**Job situation**^1^^,^^2^				
Employed/Paid Internship	602 (84.6)	188 (84.7)	414 (84.5)	
Unemployed	64 (9.0)	29 (13.1)	35 (7.1)	0.001*
Other^3^	46 (6.4)	5 (2.3)	41 (8.4)	
**Income perception**^1^^,^^2^				
Live comfortable	83 (11.6)	24 (10.8)	59 (12.0)	0.883
Can live	442 (62.0)	140 (62.8)	302 (61.6)	
Live with difficulties	188 (26.4)	59 (26.5)	129 (26.3)	
**Household Members (number)** ^1^^,^^2^				
≤3	352 (49.5)	105 (47.5)	247 (50.4)	0.769
4	283 (39.8)	91 (41.2)	192 (39.2)	
>4	76 (10.7)	25 (11.3)	51 (10.4)	
**Household classification** ^1^^,^^2^				
Mother or father with children	60 (8.4)	20 (9.0)	40 (8.2)	0.811
Couple with children	589 (82.6)	185 (83.0)	404 (82.4)	
Couple with children and other	64 (9.0)	18 (8.1)	46 (9.4)	
**Household number of children** ^1^^,^^2^				
1	356 (52.6)	95 (46.6)	270 (55.1)	0.100
2	286 (41.2)	93 (45.6)	193 (39.4)	
≥3	43 (6.2)	16 (7.8)	27 (5.5)	
**Sex of participant child**^1^				
Female	342 (47.9)	107 (47.8)	235 (48.0)	0.962
Male	372 (52.1)	117 (52.2)	255 (52.0)	
**Age of participant child**^1^				
2–3 years	322 (45.1)	95 (42.4)	227 (46.3)	0.614
4 years	224 (31.4)	73 (32.6)	151 (30.8)	
5–6 years	168 (23.5)	56 (25.0)	112 (22.9)	
**Food Insecurity level**^1^^,^^2^				
Food security	517 (77.5)	184 (85.2)	333 (73.8)	0.001*
Food insecurity	150 (22.5)	32 (14.8)	118 (26.2)	
**Nutrition knowledge**^1^^,^^2^				
≤ 50% of correct answers	104 (15.9)	24 (14.6)	80 (16.3)	
51–75% of correct answers	347 (53.1)	90 (54.9)	257 (52.4)	0.827
>75% total of correct answers	203 (31.0)	50 (30.5)	153 (31.2)	

^1^Sample size of these variables is 714 (paper: n = 224, online: n = 490), due to exclusion of cases which questionnaires were completed by other individuals than parents.

^2^ Sample size were lower due to missing values in the following variables: respondent age (n = 713, online: n = 489), job situation (n = 712, paper: n = 222), income perception (n = 713, paper: n = 223), household members (n = 711, paper: n = 221), household classification (n = 713, paper: n = 223), household number of children (n = 694, paper: n = 204), food insecurity level (n = 667, paper: n = 216; online: n = 451); nutrition knowledge (n = 654, paper: n = 164).

^3^ Other: Student, retired, disable, military/community work, domestic, other inactivity situation.

*statistically significant (p<0.05).

According to Table 1, most questionnaires were completed by mothers (82.6%), aged between 30 and 40 years old (71.9%), and highly educated (53.6%). About parent’s job situation, 84.6% of parents were employed or doing a paid activity. Related to income perception, most families reported to live without difficulties (73.6%) and approximately half of families (49.5%) had 3 or less members in their household. Regarding household characteristics, most families were constituted by both parents (82.6%) with an only child (52.6%). With regard to child’s age and sex, approximately half, were aged 4 years or older (54.9%) and female (47.9%).

A minority of families (22.5%) reported to live in a situation of food insecurity (modest, moderated or severe level). Considering families’ baseline nutrition knowledge evaluation, most parents answered correctly, at least, half of the questions (84.1%).

Regarding socio-demographic characteristics, the proportion of respondents who were fathers and, parents that reported food insecurity were higher among those who completed the online questionnaire (p = 0.001). Regarding job situation the proportion of participants who reported to be unemployed were higher among those who completed the questionnaire on paper (p = 0.001). For the other socio-demographic characteristics and for nutrition knowledge, not statistically differences were found (Table 1).

### Exploratory factorial analysis: Instrument characterization

After conducting Exploratory Factorial Analysis for the 22 items scale of parents’ practices, preferences, skills and attitudes on child’s food consumption, a total of 7 factors were extracted with major conceptual inconsistencies. After that, the Scree Plot was observed and suggested 5 factors extraction. Conceptual meaning was achieved for 5 fixed factors with a total of 21 items, Kaiser-Meyer-Olkin value of 0.770, Barttlet sphericity test was rejected (p<0.001) and an explained variance of 52.4% was observed.

Internal reliability was observed for each factor and dimensions were constructed by the mean average of items who integrated the factor (Table 2): 1. Parental modelling and active promotion of child’s F&V consumption (Reasonable Cronbach’s alpha: α = 0.73), 2. Parental skills for choosing/preparing healthy food (Reasonable Cronbach’s alpha: α = 0.75), 3. Parental food preferences and satiety perception (Reasonable Cronbach’s alpha: α = 0.70), 4. Parental awareness regarding sugar and salt intake (Weak Cronbach’s alpha: α = 0.61), and 5. Parental allowance regarding child’s F&V consumption (Weak Cronbach’s alpha: α = 0.55). The item “I can prepare meals with frozen vegetables” was not considered in the model due to its low communality value and because it was not conceptually framed in the factor where it was projected. The authors considered the item “It is healthier to my child to consume less salt” in factor 4 because it was conceptually framed, and its lower loading could be due to projection issues (high proportion of “completely agree”). In addition, internal consistency is similar with or without the item for both factor 4 and factor 1.

**Table 2:** Factors’ characterization, reliability and factorial loadings by Varimax rotation (5 factors extracted) (N = 618).

Factor/item	Paper (n = 205)	Online (n = 413)		Total participants (n = 618)	Completely agreement	Factor 1	Factor 2	Factor 3	Factor 4	Factor 5
	*Mean (SD*^*3*^*)*	*Mean (SD*^*3*^*)*	P-value^4^	*Mean (SD*^*3*^*)*	*n (%)*					
**1. Parental modelling and active promotion of child’s F&V consumption (explained variance: 21.6%; α = 0.729** ^1^**)**	**4.70 (0.39)**	**4.62 (0.49)**	**0.179**	**4.67 (0.44)**	**-**					
• We frequently eat vegetables in family	4.57 (0.71)	4.45 (0.80)	0.058	4.51 (0.75)	390 (63.1)	**0.697**				
• I encourage my child to eat vegetables	4.85 (0.37)	4.74 (0.61)	0.043*	4.79 (0.51)	508 (82.2)	**0.674**				
• We frequently eat fruit in family	4.49 (0.85)	4.46 (0.86)	0.593	4.47 (0.84)	397 (64.2)	**0.658**				
• I encourage my child to eat fruit	4.95 (0.24)	4.90 (0.34)	0.051	4.93 (0.30)	580 (93.9)	**0.605**				
• I can make my child to eat fruit as dessert	4.66 (0.64)	4.55 (0.84)	0.425	4.63 (0.73)	450 (72.8)	**0.526**				
**2. Parental skills for choosing/preparing healthy food (explained variance: 10.1%; α = 0.745)**	**4.16 (0.61)**	**4.06 (0.71)**	**0.097**	**4.10 (0.67)**	**-**					
• I can choose products with low salt content	4.13 (0.94)	3.98 (1.00)	0.056	4.07 (0.95)	237 (38.3)		**0.813**			
• I can choose products with low sugar content	4.32 (0.85)	4.16 (0.96)	0.061	4.24 (0.89)	291 (47.1)		**0.748**			
• I can prepare a pleasure meal without salt	3.53 (1.21)	3.50 (1.23)	0.731	3.52 (1.21)	141 (22.8)		**0.610**			-0.309
• I can prepare vegetable meals that my child like	4.43 (0.80)	4.25 (0.95)	0.040*	4.33 (0.88)	332 (53.7)	0.478	**0.494**			
• I can prepare meals with 1/3 of the dish with vegetables	4.38 (0.78)	4.27 (0.86)	0.145	4.33 (0.81)	309 (50.0)	0.419	**0.485**			
**3. Parental food preferences and satiety perception (explained variance: 8.1%; α = 0.702)**	**3.35 (0.92)**	**3.33 (0.99)**	**0.819**	**3.35 (0.97)**	-					
• Vegetables do not satiate me **R**^2^	3.77 (1.32)	3.65 (1.35)	0.216	3.72 (1.33)	33 (5.3)			**0.878**		
• Fruit does not satiate me **R**	3.66 (1.31)	3.62 (1.32)	0.723	3.66 (1.31)	31 (5.0)			**0.862**		
• Food without salt has no taste **R**	3.03 (1.27)	3.04 (1.35)	0.885	3.06 (1.33)	63 (10.2)			**0.608**		
• I like the sweet beverages taste **R**	2.95 (1.25)	2.92 (1.38)	0.688	2.95 (1.34)	74 (12.0)			**0.512**		
**4. Parental awareness regarding sugar and salt intake (explained variance: 7.2%; α = 0.613)**	**4.78 (0.37)**	**4.68 (0.47)**	0.034*	**4.72 (0.44)**	**-**					
• It is healthier to my child to consume less salt	4.90 (0.38)	4.81 (0.61)	0.064	4.85 (0.52)	554 (89.6)	0.418			**0.326**	
• It is important to me to avoid have sugary products easily available for my child	4.62 (0.69)	4.61 (0.73)	0.817	4.64 (0.70)	451 (73.0)				**0.675**	
• It is important to me to avoid buying food with high amount of sugar	4.72 (0.65)	4.59 (0.75)	0.009*	4.65 (0.71)	460 (74.7)				**0.674**	
• It is important that my child does not drink a high amount of sweet beverages	4.86 (0.55)	4.75 (0.75)	0.051	4.79 (0.69)	543 (87.9)				**0.658**	
• It is important to me that my child does not consume a lot of salt everyday	4.79 (0.59)	4.60 (0.93)	0.018*	4.66 (0.85)	500 (80.9)				**0.580**	
**5. Parental allowance regarding child’s F&V consumption (explained variance: 5.4%; α = 0.552)**	**4.53 (0.65)**	**4.50 (0.67)**	**0.839**	**4.52 (0.66)**	**-**					
• At home, my child can eat all the vegetables that he/she likes	4.56 (0.75)	4.51 (0.84)	0.708	4.55 (0.79)	417 (67.5)					**0.763**
• At home, my child can eat all the fruit that he/she likes	4.50 (0.77)	4.49 (0.80)	0.997	4.48 (0.80)	378 (61.2)					**0.699**
**6. I can prepare meals with frozen vegetables**	**4.20 (1.08)**	**4.26 (1.01)**	**0.662**	**4.28 (0.99)**	**-**	-	-	-	-	-

^1^ Factors’ internal reliability calculated for individuals that answered all items of each factor.

^2^ Items marked with an **R** were reverse coded.

^3^ SD–standard deviation.

^4^ P-value according to Mann-Whitney test, with 95% of confidence.

*statistically significant (p<0.05).

### Characterization of parents’ food practices, preferences, skills and attitudes on child’s food consumption

Regarding parental practices, preferences, skills and attitudes, no significant differences between participants’ responses (online vs. paper) were found for 18 items (out of 22), and for 4 dimensions (out of 5). Significant differences were observed for 4 items and 1 dimension: “I encourage my child to eat vegetables” (p = 0.043), “I can prepare vegetable meals that my child like” (p = 0.040), “It is important to me to avoid buying food with high amount of sugar” (p = 0.009) and “It is important to me that my child does not consume a lot of salt everyday” (p = 0.018), and the dimension: “Parental awareness regarding sugar and salt intake” (p = 0.034), with higher mean scores observed for participants who answered the questionnaire on paper (Table 2). Parents’ practices, preferences, skills and attitudes on child’s food consumption were evaluated considering socio-demographic characteristics and nutrition knowledge of participants (Table 3).

**Table 3:** Factors’ characterization according to socio-demographic characteristics and nutrition knowledge.

	Parental modelling and active promotion of child’s F&V consumption	Parental skills for choosing/preparing healthy food	Parental food preferences and satiety perception	Parental awareness regarding sugar and salt intake	Parental allowance regarding child’s F&V consumption	Frozen vegetables cooking skills
	Mean (SD^5^)	Mean (SD)	Mean (SD)	Mean (SD)	Mean (SD)	Mean (SD)
**Respondent**						
Mother	4.67 (0.44)	4.11 (0.65)	3.41 (0.96)	4.72 (0.44)	4.53 (0.65)	4.28 (1.02)
Father	4.63 (0.43)	4.04 (0.73)	3.05 (0.94)	4.71 (0.45)	4.45 (0.73)	4.32 (0.79)
*p*^1^	0.082	0.492	<0.001*	0.520	0.369	0.624
**Respondent age (years)** ^3^						
<30	4.41 (0.62) ^a^^6^	3.89 (0.63) ^a^	3.29 (0.89)	4.55 (0.51) ^a^	4.45 (0.65)	4.06 (1.13)
30–40	4.68 (0.42) ^b^	4.08 (0.67) ^b^	3.36 (0.97)	4.71 (0.44) ^b^	4.53 (0.66)	4.29 (0.98)
>40	4.75 (0.34) ^b^	4.26 (0.63) ^c^	3.35 (1.01)	4.82 (0.38) ^c^	4.50 (0.68)	4.36 (0.91)
*p*^2^	<0.001*	<0.001*	0.732	<0.001*	0.586	0.196
**Education level (schooling years)**						
<10	4.68 (0.37)	4.20 (0.64)	2.84 (1.06) ^a^	4.66 (0.50) ^a^	4.60 (0.57)	4.36 (1.02)
10–12	4.60 (0.51)	4.04 (0.71)	3.27 (0.98) ^b^	4.66 (0.51) ^a^	4.47 (0.68)	4.25 (1.06)
>12	4.70 (0.40)	4.11 (0.64)	3.51 (0.89) ^c^	4.76 (0.38) ^b^	4.52 (0.67)	4.29 (0.94)
*p* ^2^	0.129	0.244	<0.001*	0.047*	0.323	0.561
**Job situation**^3^						
Employed/Paid Internship	4.67 (0.43)	4.07 (0.67)	3.36 (0.95)	4.72 (0.44)	4.52 (0.67)	4.27 (0.98)
Unemployed	4.61 (0.50)	4.23 (0.66)	3.35 (1.06)	4.76 (0.40)	4.52 (0.63)	4.37 (0.96)
Other ^4^	4.71 (0.43)	4.24 (0.65)	3.22 (1.10)	4.57 (0.58)	4.56 (0.46)	4.35 (1.13)
*p* ^2^	0.456	0.075	0.817	0.294	0.885	0.449
**Income perception**^3^						
Live with difficulties	4.60 (0.46) ^a^	4.03 (0.72)	3.34 (1.00)	4.68 (0.49)	4.53 (0.63)	4.34 (0.97)
Can live	4.68 (0.43) ^b^	4.12 (0.65)	3.32 (0.97)	4.72 (0.42)	4.53 (0.64)	4.26 (1.00)
Live comfortable	4.74 (0.41) ^b^	4.12 (0.61)	3.51 (0.84)	4.77 (0.43)	4.41 (0.80)	4.25 (0.97)
*p* ^2^	0.016*	0.540	0.304	0.346	0.747	0.575
**Household Members (number)** ^3^						
≤3	4.65 (0.44)	4.08 (0.68)	3.42 (0.98)	4.70 (0.45)	4.54 (0.65)	4.23 (1.03)
4	4.67 (0.44)	4.11 (0.64)	3.27 (0.98)	4.71 (0.46)	4.50 (0.68)	4.33 (0.95)
>4	4.72 (0.45)	4.16 (0.68)	3.32 (0.83)	4.82 (0.33)	4.45 (0.62)	4.39 (0.91)
*p* ^2^	0.085	0.571	0.128	0.191	0.301	0.445
**Household classification**^3^						
Mother or father with children	4.71 (0.45)	4.21 (0.63)	3.35 (1.05)	4.67 (0.49)	4.57 (0.60)	4.26 (1.13)
Couple with children	4.66 (0.43)	4.08 (0.68)	3.34 (0.97)	4.72 (0.44)	4.52 (0.66)	4.30 (0.96)
Couple with children and other	4.65 (0.49)	4.14 (0.60)	3.41 (0.86)	4.76 (0.41)	4.41 (0.69)	4.20 (1.09)
*p* ^2^	0.687	0.381	0.991	0.642	0.365	0.885
**Household number of children**^3^						
1	4.65 (0.46)	4.09 (0.67)	3.41 (0.98)	4.71 (0.44)	4.53 (0.66)	4.24 (1.02)
2	4.68 (0.41)	4.12 (0.64)	3.31 (0.94)	4.73 (0.45)	4.50 (0.66)	4.38 (0.91)
≥3	4.69 (0.50)	4.06 (0.82)	3.05 (0.97)	4.71 (0.45)	4.56 (0.47)	4.25 (1.02)
*p* ^2^	0.213	0.887	0.084	0.674	0.654	0.277
**Sex of participant child**						
Female	4.70 (0.42)	4.16 (0.66)	3.40 (0.97)	4.74 (0.45)	4.53 (0.65)	4.30 (0.95)
Male	4.64 (0.45)	4.04 (0.67)	3.30 (0.96)	4.69 (0.44)	4.50 (0.67)	4.27 (1.02)
*p* ^1^	0.178	0.013*	0.180	0.027*	0.668	0.910
**Age of participant child (years)**						
2–3	4.66 (0.46)	4.09 (0.67)	3.33 (0.94)	4.70 (0.45)	4.57 (0.58)	4.30 (0.97)
4	4.65 (0.42)	4.09 (0.66)	3.36 (0.96)	4.73 (0.47)	4.42 (0.72)	4.25 (0.98)
5–6	4.70 (0.43)	4.12 (0.67)	3.37 (1.02)	4.73 (0.41)	4.55 (0.70)	4.29 (1.01)
*p* ^2^	0.280	0.890	0.859	0.282	0.061	0.797
**Food Insecurity level** ^3^						
Food security	4.68 (0.44)	4.09 (0.68)	3.34 (0.94)	4.75 (0.39)	4.53 (0.64)	4.29 (0.98)
Food insecurity	4.66 (0.42)	4.14 (0.63)	3.41 (1.05)	4.74 (0.39)	4.52 (0.73)	4.30 (1.02)
*p* ^1^	0.410	0.642	0.312	0.636	0.414	0.616
**Nutrition knowledge** ^3^						
≤ 50% of correct answers	4.60 (0.48) ^a^	4.16 (0.70)	3.04 (1.02) ^a^	4.57 (0.57) ^a^	4.46 (0.67)	4.35 (0.90)
51–75% of correct answers	4.64 (0.47) ^a,b^	4.05 (0.70)	3.36 (0.97) ^b^	4.72 (0.43) ^a,b^	4.50 (0.69)	4.28 (0.98)
>75% total of correct answers	4.72 (0.39) ^b^	4.15 (0.61)	3.55 (0.90) ^c^	4.76 (0.42) ^b^	4.55 (0.63)	4.33 (0.98)
*p* ^2^	0.041*	0.377	<0.001*	0.038*	0.656	0.739

^*1*^ *p* value calculated according with Mann-Whitney test, with 95% of confidence.

^2^ *p* value calculated according with Kruskal-Wallis test, with 95% of confidence.

^3^ Sample size were lower due to missing values in the following variables: Respondent age (n = 617), job situation (n = 617), income perception (n = 617), household members (n = 616), household classification (n = 617), household number of children (n = 599), food insecurity (n = 583), nutrition knowledge (n = 564).

^4^ Other: Student, retired, disable, military/community work, domestic, other inactivity situation.

^5^ SD–standard deviation.

^6^ a,b,c homogeneous subsets according to Mann-Whitney test, with 95% of confidence.

*statistically significant (p<0.05).

Mothers revealed a significantly more positive perception of F&V’s satiety ability than fathers and less appetite for salt and sweet beverages (p<0.001).

Parents aged 30 or more years old, revealed to have significantly more influence on child´s behaviors modulation and active promoting of F&V consumption than younger parents (p<0.001). Also, considering skills for choosing/preparing healthy food and awareness regarding sugar and salt intake, older fathers or mothers had significantly higher skills (p<0.001) and higher concerns regarding salt and sugar intake (p<0.001) than younger ones.

Parents from the higher education groups had a significantly more positive perception of F&V’s satiety ability, and less appetite for salt and sweet beverages than their peers from lower education groups (p<0.001). Parental awareness regarding sugar and salt intake was also significantly higher in the group with more than 12 schooling years compared to lower education groups (p = 0.047).

Regarding parental income perception, parental modelling and active promotion of F&V consumption were significantly lower in families who reported to live with difficulties comparing to families who live without constraints (p = 0.016).

It was observed that families with a participant female child, had significantly higher skills for choosing/preparing healthy food (p = 0.013) and higher concerns regarding sugar and salt intake comparing with families with a participant male child (p = 0.027).

Parents who revealed lower nutrition knowledge (≤ 50% of correct answers), showed to have fewer practices at home related to positive parental modelling and active promotion of F&V consumption (p = 0.041) and minor concerns regarding child’s sugar and salt intake (p = 0.038), comparing with parents who revealed higher nutrition knowledge at baseline (>75% of correct answers). A significantly better perception of F&V’s satiety ability and lower appetite for salt and sweet beverages was observed for parents with higher baseline nutrition knowledge (p<0.001).

For parental allowance regarding F&V consumption and frozen vegetables, significant differences were not identified according to socio-demographic characteristics. In general, the lowest mean score was obtained for the parental perception of F&V satiety and preference for salt/sugar flavor. It was observed that a high percentage of parents considered that food without salt has no taste and revealed to like sweet beverages taste.

## Discussion

### Parents’ practices, preferences, skills and attitudes on child’s food consumption assessment: Instrument proprieties

In the present study, we tested a questionnaire developed to assess parental practices, preferences, skills and attitudes regarding food consumption. Five dimensions were identified, and one item was excluded (a total of 21 items). Internal reliability of all factors was acceptable (calculated through Cronbach’s alpha), showing satisfying properties. Some dimensions showed low levels of internal reliability possible due to the reduced number of items.

This study shows that the holistic, multidimensional scale studied, easily administrated, may have a high potential for the study of parents’ practices, preferences, skills and attitudes. However, the analytic ability of scale may be improved.

Our results showed that dimensions could interact in a predictable or non-predictable way, reflecting the complexity of parental influences on child’s food consumption. Frozen vegetables were understood by parents as a specific construct apart from fresh vegetables, which may be related to the perceived lower nutritional value of frozen vegetables, comparing to fresh vegetables. This item was not included neither in parental skills for choosing/preparing healthy food nor in parental modelling and active promotion of child’s F&V consumption, as expected, revealing to be a dimension itself. These results are in line with other studies which showed that individuals have an implicit negative association with frozen vegetables, comparing to fresh vegetables [44] and that consumers associates a higher level of freshness of F&V with minimal product transformation, among other characteristics [45]. Considering this specific theme as a dimension itself, an improvement of this dimension might be explored, namely through a more complex and complete evaluation, including parental perception of frozen vegetables quality and healthiness.

Some differences were observed when comparing dimensions predicted *apriori*, with final dimensions proposed by the model. According to EPHE [46], items related to energy balance-related behaviors (“We frequently eat vegetables in family” / “We frequently eat fruit in family”) were considered in a different dimension than items evaluating active promoting (“I encourage my child to eat vegetables” /”I encourage my child to eat fruit”), which were grouped in the same dimension in our study. Our results confirm that parental allowance and active promotion have distinguished behavioral basis and different levels of parental permissiveness and involvement [47, 48]. To allow a more complete evaluation of parental allowance about child’s eating habits, we consider it should be also evaluated the parental permissiveness to their child to eat energy-dense foods, with high amounts of sugar and salt. This questionnaire shows satisfying properties to investigate several parental practices, preferences, skills and attitudes on child’s food consumption, of F&V, sugar and salt intake.

### Characterization of parents’ food practices, preferences, skills and attitudes on child’s food consumption

In the present study, it was observed that mothers, older parents, higher educated, those who reported living with no difficulties regarding household income, parents of female children and with higher nutrition knowledge revealed to have significantly higher scores for different dimensions assessed.

Specifically, fathers showed considerable worst ability to identify satiety of F&V, and higher appetite for sugar beverages and salt’s flavor than mothers. These results are consistent with previous findings reported in the literature, which showed that women demonstrated healthier habits [32] and mothers were more likely to be in a healthy weight than fathers [49]. According to a national survey, the average sodium intake was excessive and higher in men compared to women [4] which are in line with our findings.

On the other hand, it would also be expected to observe higher monitoring and concern for children’s eating habits among mothers [50–52], along with higher awareness of the importance of healthy eating [32]. However, in our study, differences between mothers and fathers in those dimensions were not statistically significant.

With increasing age, it was observed an increased parental promotion of child’s F&V consumption through active encouragement and modelling, along with higher awareness regarding sugar and salt intake. These findings are consistent with other studies where healthy eating habits and more concerns about health increases with age [53–55]. Younger parents demonstrated the worst practices and concern about their child’s eating habits which suggests that this group may benefit from an intervention to improve their children’s eating habits.

Another important socio-demographic variable associated with parental practices described in literature, is education [56–58]. In this work, parents less educated revealed to have higher preference for sugar/salt and less perception of F&V satiety ability and lower awareness level regarding child’s sugar and salt intake. These findings are in line with studies showing that education level is associated with better health habits, namely, higher F&V consumption [6]. Other authors reported the impact of higher educated parents in child’s fruit consumption [55] and less sugar-sweetened beverages intake [59]. Parental education is an important determinant of their eating habits, which in turn, influences what children eat [60]. Higher awareness is also consistent with other studies showing that education level is inversely associated with the use of food as rewards [61, 62]. Our results suggest that interventions promoting healthier eating habits are needed and must specially address less educated parents, who may benefit of a better understanding of sugar and salt’s consequences on health and to be aware of the feasibility of preparing tasty food with no salt addiction.

Along with education level, income has also been recognized as one of the most important determinants of eating habits and health [55]. In our study, parents who reported to live with difficulties demonstrated to have worst practices regarding F&V consumption and be less effective in managing child’s F&V intake comparing with families from higher income. This relation gains relevance considering parent role modeling [60], and it is known that families from low socioeconomic status have worse habits, both adults and children [55]. Considering that most children do not like F&V taste [63] and do not meet its recommendations [64], parental involvement is necessary. Previous results highlighted the need for understanding possible constraints of families related to socioeconomic status and interventions should try to overcome them for positive results.

Differences were found in parental skills for cooking and product selection and awareness regarding sugar/salt intake, according to children’s sex. Parents demonstrated higher scores for these dimensions with their daughters, comparing with parents with a male child. This association was consistent with other studies revealing that parents use different parenting styles for their sons and daughters [65]. It has been reported that children’s sex influences parents’ concerns about weight [66]. M. Campbell, J. Williams [67] demonstrated that mothers were more concerned about weight/health issues of their daughters than with their sons. Despite that, boys are more likely to consume energy-dense foods [4, 68] which may indicate their need for higher monitoring than girls.

Furthermore, it is known that nutrition knowledge is directly associated with eating habits [25, 69]. Parents with higher nutrition knowledge showed higher scores for parental modelling and active promotion of F&V consumption. Our findings are consistent with previous studies that demonstrated an association between nutrition knowledge and eating habits [70, 71]. According to Tabbakh and Freeland-Graves [72], higher maternal nutrition knowledge was found to be related with higher fruit consumption of their child and better quality of overall diet. Similar results were also found with regard to awareness for sugar and salt intake. More knowledge was found to be a good indicator of higher awareness for salt disadvantages [73] and healthier behavior adoption seems to depend of profound knowledge of the health issue. Better food preferences were also found in parents with higher nutrition knowledge, and once more, this association is consistent with previous findings. Nutrition education revealed to predict and increase F&V consumption in overweight adults [69], and increasing knowledge about sugar, was associated with a reduction in sugary food and beverages consumption [74]. Also, in children, F&V consumption was higher in individuals with higher nutrition knowledge [75].

### Limitations and future recommendations

Interpretation of these results must consider study limitations. A convenience sample was used in this study. Respondents were mostly mothers, employed, highly educated, in a food security situation, and most individuals revealed to be conscientious about all dimensions and to have reasonable nutrition knowledge. Thus, the socio-demographic characteristics of our sample are different comparing to general population. However, this study had a wide geographical distribution, which may overcome the use of a convenience sample. Another limitation is the use of two distinguished methods of data collection, which resulted in some minor but statistically significant differences between participants who respond the questionnaire online or on paper. Nevertheless, these differences could be related to the small sample size. Higher scores for participants who answered the paper version of the questionnaire could be due to differences in individuals’ profiles or dependent of the format itself. Different tools needed for completing the questionnaire on paper (pen) or online (computer, internet) could influence the surrounding environment in which the questionnaire was fulfilled, and the time spent for completing the questionnaire, which may in turn, conditioning participants’ answers.

The instrument developed and tested under this study showed satisfying characteristics that allow us to investigate parental influences (practices, preferences, skills and attitudes) on child’s food consumption regarding F&V, sugar and salt intake. This instrument reveals to have the advantage of being short and easily delivered, allowing at the same time a comprehensive evaluation on a broad range of parental influences. Future studies using this reliable instrument, focusing on the evaluation of parental habits and influences on child’s intake of F&V, sugar and salt, should be performed in representative samples, including both individuals from high and low socioeconomic status and educational levels. Studies may benefit from a deeper evaluation of parents’ perception of frozen vegetables and allowance practices regarding sugar and salt intake. For more reliable information, an adequate method of data collection should be defined *apriori* based on the resources and socio-demographic characteristics of the population, which were emphasized to be important.

## Supporting information

S1 File (RAR)

S2 File (DOCX)

S3 File (DOCX)
