# Supplementary material for: Parental practices, preferences, skills and attitudes on food consumption of pre-school children: Results from Nutriscience Project
Source: PLoS One. 2021 May 25;16(5):e0251620. doi: 10.1371/journal.pone.0251620 (PMC8148319; doi:10.1371/journal.pone.0251620)
Supplement: S2 File — (DOCX) [file pone.0251620.s002.docx]

**PARENTAL QUESTIONNAIRE**

**NUTRITION LITERACY ASSESSESMENT**

**Welcome to the Nutriscience Project!**

The Nutriscience project aims to increase nutrition literacy of participants in a playful and interactive way.

We ask that you provide the information as much reliable as possible, having in mind that your responses are completely confidential.

This questionnaire consists on two groups of questions, the first on general data about sociodemographic characteristics and the second focusing on food and nutrition knowledge.

The response time is approximately 10 minutes.

Thank you for your interest and collaboration.

**Sociodemographic characteristics**

| 1. Indicate the number of household members, including yourself. |
| --- |
| **Number of household members: ______________** |

| 1. **This questionnaire will be completed by (a):** | 1. **How old are you? (the person who is answering to the questionnaire)** |
| --- | --- |
| - Mother - Father - Brother / sister - Grandfather / Grandmother - Other: _____________________ | **Age: ______________ years old** |

**3.2 – Household number of children: ______________________**

| - 1. How do you classify your household? |
| --- |
| - Only a mother or a father with child/children (monoparental family) - Couple with children - Couple with children and other |

| **4. Regarding your child that attend the participant school in the project, please tell us:** | | | | |
| --- | --- | --- | --- | --- |
| **4.1 Child’s sex:** | | | **4.2 Child’s age:** | |
| - Male - Female | | **Age: ______________** years old | | |
| 1. **Indicate your education level and your spouse’s education level:** | | | | |
| - 1. About yourself: | 5.2 About your spouse: | | |  |
| - Elementary school (1^st^ to 4^th^ year) - Middle school (5^th^ to 6^th^ year) - Middle school (7^th^ to 9^th^ year) - High school (10^th^ to 12^nd^ year) - University degree | - Elementary school (1^st^ to 4^th^ year) - Middle school (5^th^ to 6^th^ year) - Middle school (7^th^ to 9^th^ year) - High school (10^th^ to 12^nd^ year) - University degree - I don’t have a spouse | | |  |
| 1. **What is your professional situation? And your spouses’ professional situation?** | | | | |
| - 1. About yourself: | 6.2 About your spouse: | | |  |
| - Employed or doing a paid job - Unemployed - Student or unpaid professional activity - Retired or early retired - Disable - Performing military service or community work - Domestic/Housework - Other inactivity situation | - Employed or doing a paid job - Unemployed - Student or unpaid professional activity - Retired or early retired - Disable - Performing military service or community work - Domestic/Housework - Other inactivity situation - I don’t have a spouse | | |  |

| **7. In what type of company do you work? And your spouse?** | | | |
| --- | --- | --- | --- |
| 7.1. About yourself: | 7.2 About your spouse: | | |
| - Central and local public administration - Other services belonging to the public function (education and health) - Public company - Private company - Self-employment - Other | - Central and local public administration - Other services belonging to the public function (education and health) - Public company - Private company - Self-employment - Other - I don’t have a spouse | |  |
|  | |  | |

| 1. **Which of the following descriptions is most closely to the way you feel these days about your household income?** |
| --- |
| - Live comfortable with current income - I can live with current income - It is difficult to live with current income - It is very difficult to live with current income |

**Nutrition literacy**

**I. NUTRIENTS**

Nutrients, compounds that are present in food, are essential for the growth and adequate functioning of the organism. The following questions are related to nutrients and their food sources in our diet. From the following options, select the option that you consider most correct to complete the sentence:

1. The starch in a slice of bread is a type of _.

A. fat

B. vitamin

C. carbohydrate

D. protein

2. Olive oil and butter are a food source of .

A. vitamin C

B. carbohydrate

C. iron

D. fat

3. The present in Orange juice is a type of carbohydrate.

A. sugar

B. calcium

C. iron

D. folate

4. Eggs, chicken and fish are good food-sources of ____________.

A. amido

B. proteína

C. fibra

D. açúcar

5. A spinach soup is a good food-source of: .

A. fiber

B. fat

C. calories

*D.* protein

6. From the following options, indicate the one that you consider to have high amount of sodium .

A. 2 sausages

B. 1 chocolate

C. 1 bread

D. 4 water and salt biscuit

**II. FOOD PORTIONS**

Below are presented some photographs of meal dishes with the various components that usually form part of a main meal (lunch and dinner). From the following options, select the option that you consider is the most adequate.

| 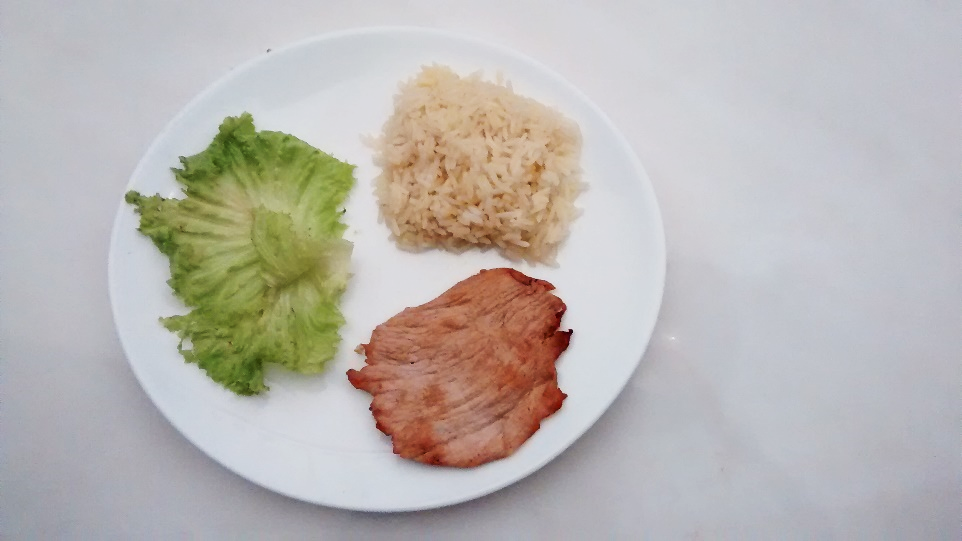 | 1. From the following meal dish, what is not right?  A. Have a small portion of meat  B. Have a small portion of vegetables  C. Have a small portion of rice  E. It is all right; it is the ideal dish |
| --- | --- |
| 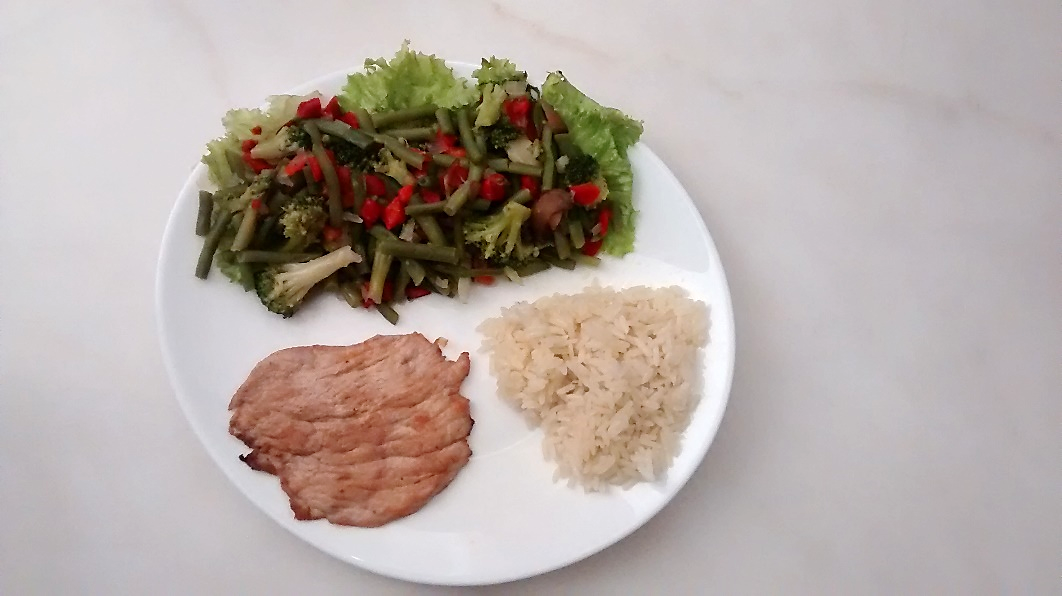 | 2. From the following meal dish, what is not right?  A. Have a small portion of meat  B. Have a large portion of vegetables  D. Have a small portion of rice  E. It is all right; it is the ideal dish |
| 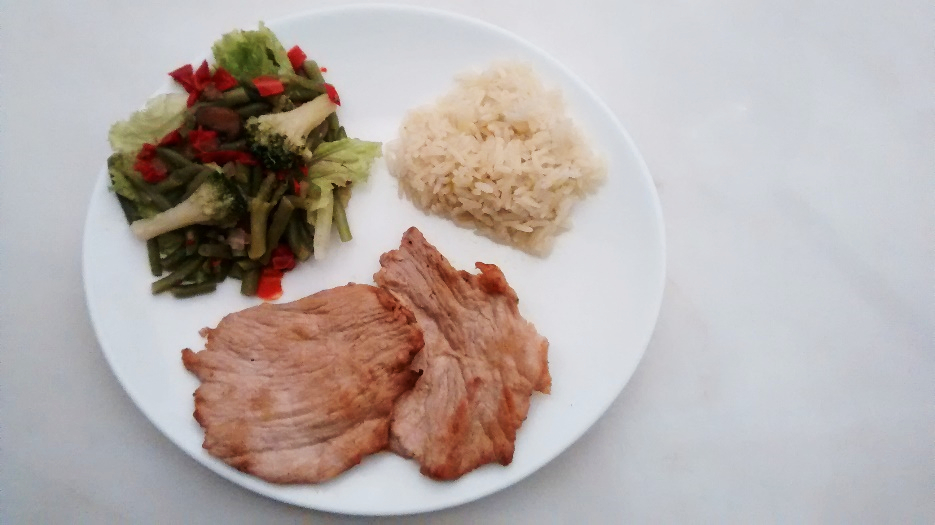 | 3. From the following meal dish, what is not right?  A. Have a large portion of meat  B. Have a large portion of vegetables  C. Have a small portion of rice  E. It is all right; it is the ideal dish |

**III. FOOD GROUPS**

1. According to the recommendations of the Portuguese Food Wheel Guide, different food groups should be consumed in different amounts. Please order the food groups belonging to Portuguese Food Wheel Guide, from 1 to 7 (1- those that should be consumed in daily larger amounts and 7 - those that should be consumed in daily smaller amounts).

Meat, fish and eggs _______

Cereals and Tubers _______

Fruits _______

Vegetables _______

Milk and dairy products _______

Pulses _______

Oils and fats _______

**IV. FOOD LABELS**

1. The following food labels represents two diferent yogurts: **Yogurt A** and **Yogurt B**.
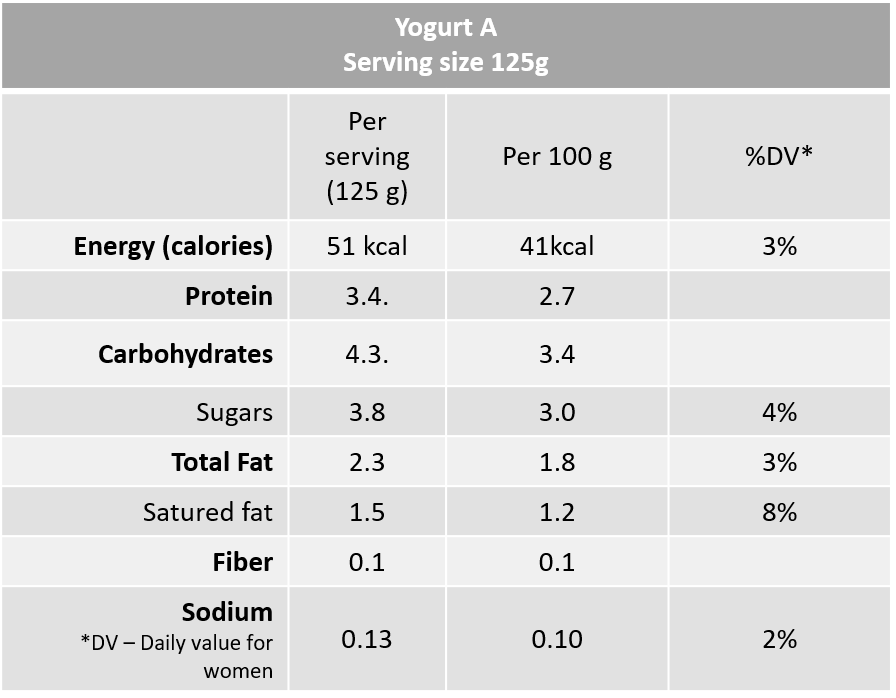


**
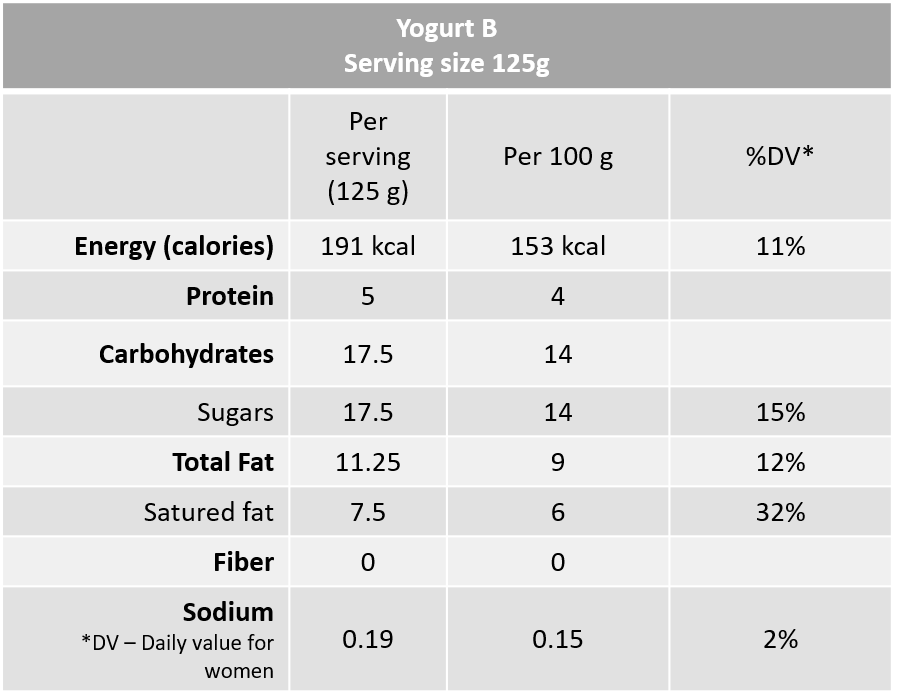
**

1.1 By analyzing the two labels, which do you consider to be the healthiest yogurt?

A. Yogurt A

B. Yogurt B

- 1. Which were the labels’ nutritional parameters that you had focus to make your choice?

| - Energy (calories) - Protein - Carbohydrates - Sugar | - Total fat - Satured fat - Fiber - Sodium | |  |
| --- | --- | --- | --- |
|  | |  | |

1. The following list of ingredients belongs to a label of a cookies’ package.

| **Cookies A** |
| --- |
| **Ingredients:** flour mixture (wheat, rice, rye, barley and oat), oat flakes, sunflower vegetable oil, sugar, invert sugar, glucose syrup, wheat bran, raising agents (E500i, E503i), salt, emulsifiers (E322), whey powder, antioxidants (E304i, tocopherol-rich extract) |

- 1. From the following ingrediente sets, please identify the one that corresponds to sugar.

| - Sugar, inverted sugar and antioxidants - Sugar and inverted sugar - Sugar, inverted sugar and glucose syrup - Sugar - Sugar, inverted sugar and emulsifiers |  |
| --- | --- |

- 1. By the analysis of previous list of ingredients of cookies, which ingredient is present in the highest amount?

| - Oat flakes - Sugar - Inverted sugar - Wheat bran - Emulsifiers - Whey powder | - Flour mixture - Sunflower vegetable oil - Glucose syrup - Raising agents - Salt - Antioxidants |
| --- | --- |

**V. ATTITUDES, PRACTICES, SKILLS AND FOOD PREFERENCES**

For the following statements, indicate your degree of agreement with them (strongly agree, partially agree, neither agree nor disagree, partially disagree, strongly disagree).

|  | Strongly agree | Partially agree | Neither agree nor disagree | Partially disagree | Strongly disagree | I do not want to answer |
| --- | --- | --- | --- | --- | --- | --- |
| 1. I encourage my child to eat fruit |  |  |  |  |  |  |
| 1. At home, my child can eat all the fruit that he/she likes |  |  |  |  |  |  |
| 1. We frequently eat fruit in family |  |  |  |  |  |  |
| 1. Fruit does not satiate me |  |  |  |  |  |  |
| 1. Vegetables do not satiate me |  |  |  |  |  |  |
| 1. I encourage my child to eat vegetables |  |  |  |  |  |  |
| 1. We frequently eat vegetables in family |  |  |  |  |  |  |
| 1. At home, my child can eat all the vegetables that he/she likes |  |  |  |  |  |  |
| 1. It is important to me that my child does not consume a lot of salt everyday |  |  |  |  |  |  |
| 1. It is healthier to my child to consume less salt |  |  |  |  |  |  |
| 1. Food without salt has no taste |  |  |  |  |  |  |
| 1. It is important that my child does not drink a high amount of sweet beverages |  |  |  |  |  |  |
| 1. I like the sweet beverages taste |  |  |  |  |  |  |
| 1. It is important to me to avoid buying food with high amount of sugar |  |  |  |  |  |  |
| 1. It is important to me to avoid have sugary products easily available for my child |  |  |  |  |  |  |
| 1. I can make my child to eat fruit as dessert |  |  |  |  |  |  |
| 1. I can prepare meals with frozen vegetables |  |  |  |  |  |  |
| 1. I can prepare meals with 1/3 of the dish with vegetables |  |  |  |  |  |  |
| 1. I can prepare vegetable meals that my child like |  |  |  |  |  |  |
| 1. I can choose products with low salt content |  |  |  |  |  |  |
| 1. I can prepare a pleasure meal without salt |  |  |  |  |  |  |
| 1. I can choose products with low sugar content |  |  |  |  |  |  |

**Food insecurity**

**It is known that the price of food can influence the choices we make. The following questions are related to the influence that economic issues can have on eating habits. For each of the following questions, answer ‘yes’ or ‘no’.**

| **Question** | **No** | **Yes** | **I do not want to answer** |
| --- | --- | --- | --- |
| 1. In the past three months, have you been concerned that your food will end before you have the money to buy more food? |  |  |  |
| 1. In the past three months, did you run out of food before you had the money to buy more food? |  |  |  |
| 1. In the past three months, has your household run out of money to have a healthy and varied diet? |  |  |  |
| 1. In the past three months, did members of your household eat only a few types of food they still had because the money ran out? |  |  |  |
| 1. In the past three months, has an adult in your household missed a meal because there was no money to buy food? |  |  |  |
| 1. In the past three months, did any adult in your household eat less than you thought you should because there was no money to buy food? |  |  |  |
| 1. In the past three months, did any adult in your household feel hungry, but did not eat because they did not have the money to buy food? |  |  |  |
| 1. In the past three months, did any adult in your household spent a whole day without a meal, or did they only have one meal during a day because they did not have the money to buy food? |  |  |  |
| 1. In the past three months, have children in your household been unable to eat healthy and varied diet because there was no money to buy food? |  |  |  |
| 1. In the past three months, did the children in your household eat only a few types of food that were still in this household because the money ran out? |  |  |  |
| 1. In the past three months, did any child in your household eat less than you thought they should because there was no money to buy food? |  |  |  |
| 1. In the past three months, has the amount of food decreased from any child’s meals in your household since there was not enough money to buy the food? |  |  |  |
| 1. In the past three months, have any children in your household missed a meal because there was no money to buy food? |  |  |  |
| 1. In the past three months, did any child in your household feel hungry but did not eat because there was no money to buy more food? |  |  |  |
