# Supplementary material for: Parental practices, preferences, skills and attitudes on food consumption of pre-school children: Results from Nutriscience Project
Source: PLoS One. 2021 May 25;16(5):e0251620. doi: 10.1371/journal.pone.0251620 (PMC8148319; doi:10.1371/journal.pone.0251620)
Supplement: S3 File — (DOCX) [file pone.0251620.s003.docx]

**QUESTIONÁRIO AOS PAIS**

**PARA AVALIAÇÃO DA LITERACIA NUTRICIONAL E ALIMENTAR**

**Bem-vindo ao Projeto Nutriciência!**

O projeto Nutriciência tem como objetivo aumentar os conhecimentos de nutrição e alimentação de uma forma lúdica e interativa.

Pedimos que forneça a informação o mais fidedigna possível tendo em conta que as suas respostas são completamente confidenciais.

Este questionário é composto por dois grupos de questões, o primeiro relativo a dados gerais da sua família e o segundo relativo aos conhecimentos de alimentação e nutrição.

O tempo de resposta é de aproximadamente 10 minutos.

Obrigada pelo seu interesse e colaboração.

**Dados Sociodemográficos**

| 1. Indique o número de pessoas que residem habitualmente na sua casa, incluindo o próprio. |
| --- |
| **Número de pessoas: ______________** |

| 1. **Este questionário vai ser preenchido pelo (a):** | 1. **Qual a sua idade? (da pessoa que está a responder)** |
| --- | --- |
| - Mãe - Pai - Irmão / Irmã - Avô / Avó - Outro: _____________________ | **Idade: ______________** anos |

**3.2 - Número de filhos no agregado familiar: ______________________**

| - 1. Como classifica o seu agregado familiar? |
| --- |
| - Só mãe ou pai com filhos (família monoparental) - Casal com filhos - Casal/Mãe/Pai com filhos e outras pessoas (família alargada) |

| **4. Relativamente ao seu filho(a) que frequenta esta Misericórdia, diga-nos por favor:** | | | | |
| --- | --- | --- | --- | --- |
| **4.1 Qual o género?** | | | **4.2 Qual a idade?** | |
| - Masculino - Feminino | | **Idade: ______________** anos | | |
| 1. **Qual o seu nível de escolaridade? E do seu cônjuge/companheiro?** | | | | |
| - 1. O próprio | 5.2 Cônjuge/Companheiro | | |  |
| - 1º ciclo do ensino básico (1º ao 4º ano) - 2º ciclo do ensino básico (5º ao 6º ano) - 3º ciclo do ensino básico (7º ao 9º ano) - Ensino secundário (10º ao 12º ano) - Ensino superior | - 1º ciclo do ensino básico (1º ao 4º ano) - 2º ciclo do ensino básico (5º ao 6º ano) - 3º ciclo do ensino básico (7º ao 9º ano) - Ensino secundário (10º ao 12º ano) - Ensino superior - Não tenho cônjuge/ companheiro | | |  |
| 1. **Como define a sua atual situação face ao trabalho? E a do seu cônjuge/companheiro?** | | | | |
| - 1. O próprio | 6.2 Cônjuge/companheiro | | |  |
| - Exerce um trabalho ou profissão, incluindo um estágio remunerado. - Desempregado - Estudante ou a frequentar outro tipo de formação ou experiência profissional não remunerada - Reformado ou reforma antecipada - Permanentemente incapacitado - A cumprir serviço militar ou trabalho comunitário - Ocupa-se das tarefas domésticas - Outra situação de inatividade | - Exerce um trabalho ou profissão, incluindo um estágio remunerado. - Desempregado - Estudante ou a frequentar outro tipo de formação ou experiência profissional não remunerada - Reformado ou reforma antecipada - Permanentemente incapacitado - A cumprir serviço militar ou trabalho comunitário - Ocupa-se das tarefas domésticas - Outra situação de inatividade - Não tenho cônjuge/companheiro | | |  |

| **7. Em que tipo de empresa/instituição trabalha? E o seu cônjuge/companheiro?** | | | |
| --- | --- | --- | --- |
| 7.1. O próprio | 7.2 Cônjuge/companheiro | | |
| - Administração pública central e local - Outros serviços pertencentes à função pública (ex: área de educação e saúde) - Empresa pública - Empresa privada - Trabalho por conta própria - Outro | - Administração pública central e local - Outros serviços pertencentes à função pública (ex: área de educação e saúde) - Empresa pública - Empresa privada - Trabalho por conta própria - Outro - Não tenho cônjuge/companheiro | |  |
|  | |  | |

| 1. **Quais das descrições seguintes mais se aproxima da forma como se sente, nos dias de hoje, em relação ao rendimento do seu agregado familiar?** |
| --- |
| - Vivo confortavelmente com o atual rendimento - Consigo viver com o atual rendimento - É difícil conseguir viver com o atual rendimento - É muito difícil conseguir viver com o atual rendimento |

**Literacia alimentar e nutricional**

1. **NUTRIENTES**

Os nutrientes, compostos que constituem os alimentos, são essenciais para o crescimento e bom funcionamento do organismo. As perguntas que se seguem têm a ver com os nutrientes e com as suas fontes na nossa alimentação. Das seguintes opções, selecione a opção que considera mais correta para completar a frase.

1. O amido que existe numa fatia de pão é um tipo de _.

A. gordura

B. vitamina

C. hidrato de carbono

D. proteína

2. Alimentos como o azeite e a manteiga são uma fonte de .

A. vitamina C

B. hidratos de carbono

C. ferro

D. gordura

3. O encontrado no sumo de laranja natural é um tipo de hidrato de carbono.

A. açúcar

B. cálcio

C. ferro

D. folato

4. Alimentos como ovos, frango e peixe são boas fontes de ____________.

A. amido

B. proteína

C. fibra

D. açúcar

5. Uma sopa de espinafres é rica em .

A. fibra

B. gordura

C. calorias

*D.* proteína

6. Das opções seguintes a que tem maior quantidade de sódio é .

A. 2 salsichas

B. 1 chocolate

C. 1 pão

D. 4 bolachas de água e sal.

**II. PORÇÕES DE ALIMENTOS**

De seguida são apresentadas algumas fotografias de pratos de refeição com os diversos componentes que habitualmente fazem parte de uma refeição principal (almoço e jantar). Das seguintes opções, selecione a opção que considera mais correta.

| 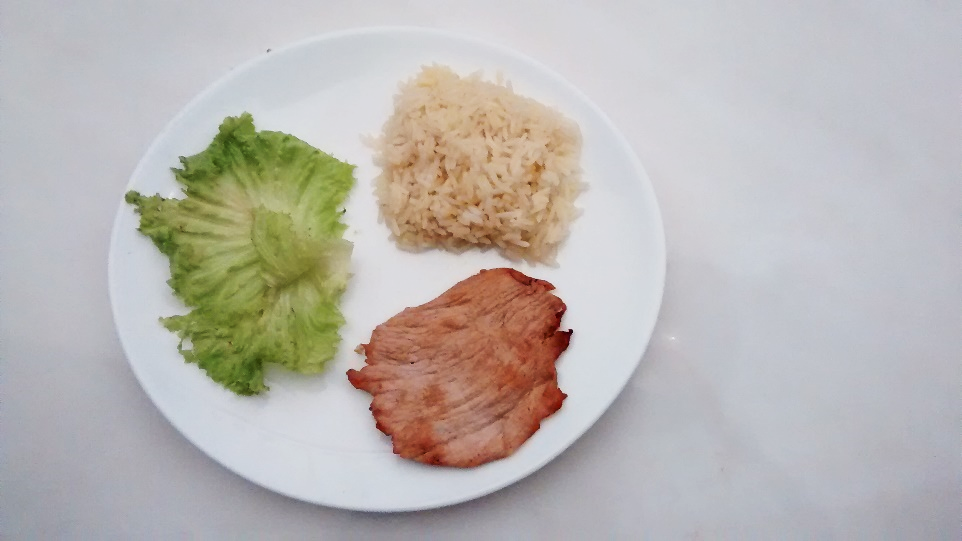 | 1. No prato apresentado na imagem, o que é que não está bem?  A. Tem pouca carne  B. Tem poucos vegetais  C. Tem pouco arroz  E. Está tudo bem; é o prato ideal. |
| --- | --- |
| 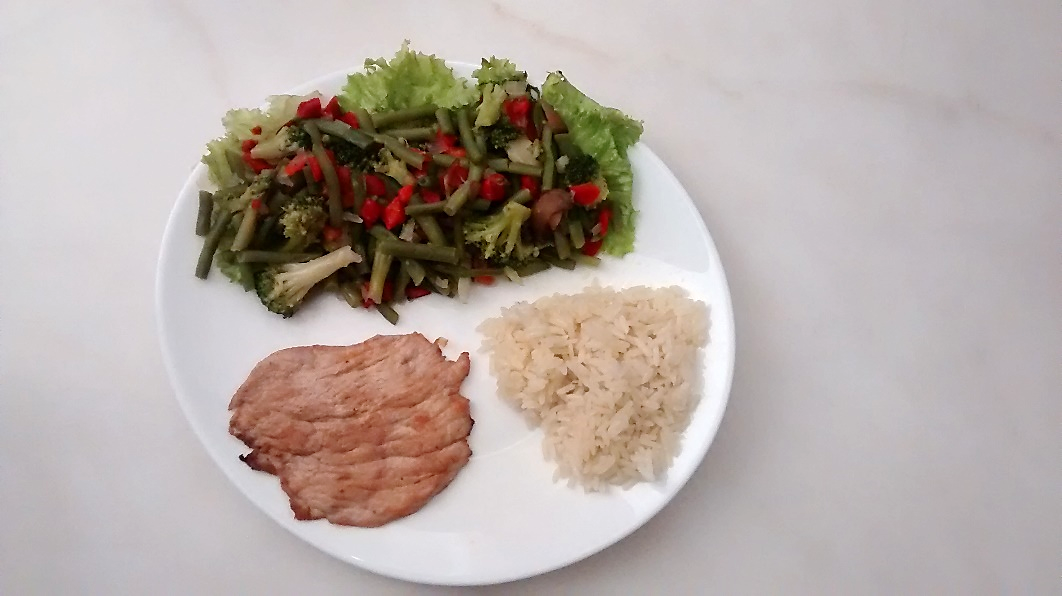 | 2. No prato apresentado na imagem, o que é que não está bem?  A. Tem pouca carne  B. Tem muitos vegetais  D. Tem pouco arroz  E. Está tudo bem; é o prato ideal. |
| 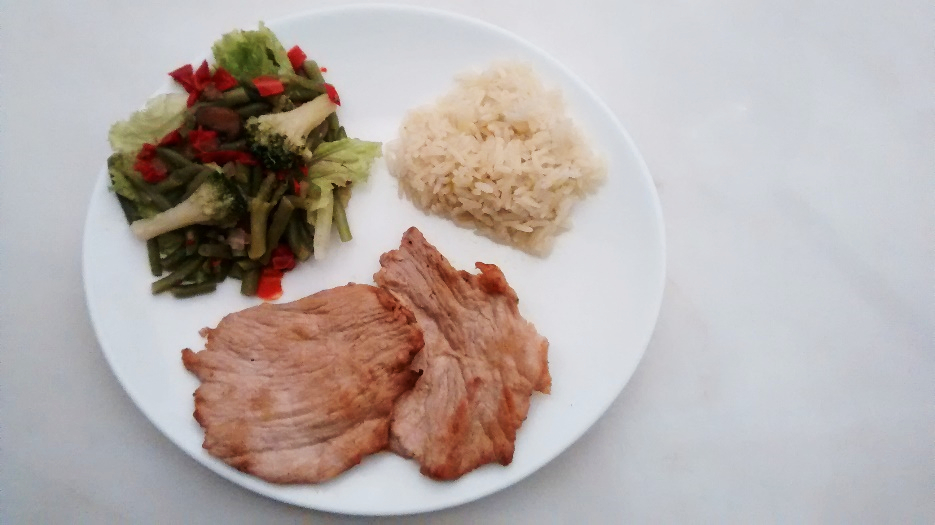 | 3. No prato apresentado na imagem, o que é que não está bem?  A. Tem muita carne  B. Tem poucos vegetais  C. Tem pouco arroz  E. Está tudo bem; é o prato ideal |

**III. GRUPOS DE ALIMENTOS**

1. De acordo com as recomendações da Roda dos Alimentos, os diferentes grupos de alimentos devem ser consumidos em diferentes quantidades. Ordene os grupos da Roda dos Alimentos, dos que devem ser consumidos em menor quantidade até aqueles que devem ser consumidos em maior quantidade diariamente. (1 em maior quantidade, 7 em menor quantidade).

Carne, pescado e ovos _______

Cereais e Tubérculos _______

Frutos _______

Hortícolas _______

Lacticínios _______

Leguminosas _______

Óleos e Gorduras _______

**IV. RÓTULOS DE ALIMENTOS**

1.
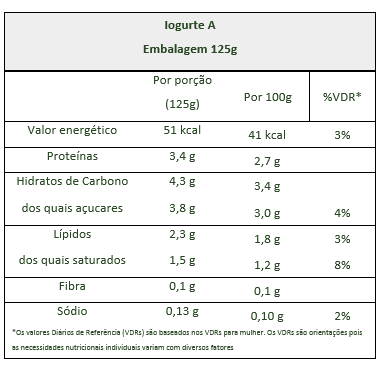

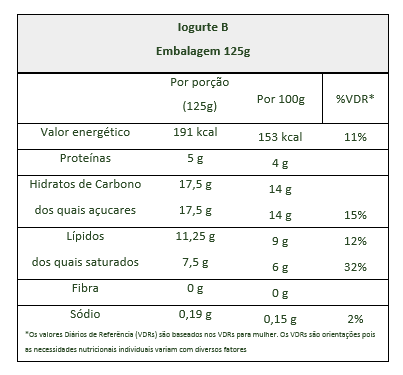
Os rótulos apresentados pertencem a dois iogurtes diferentes: **iogurte A** e **iogurte B**

Analisando os rótulos das duas embalagens, indique se as frases são verdadeiras ou falsas.

1.1 Analisando os rótulos das duas embalagens, qual considera ser o iogurte mais saudável?

A. Iogurte A

B. Iogurte B

- 1. Quais foram os parâmetros do rótulo em que se baseou para fazer a sua escolha?

| - Valor Energético - Proteínas - Hidratos de Carbono - Açúcares | - Lípidos - Lípidos Saturados - Fibra - Sódio | |  |
| --- | --- | --- | --- |
|  | |  | |

1. A lista de ingredientes apresentada pertence ao rótulo de um pacote de **bolachas.**


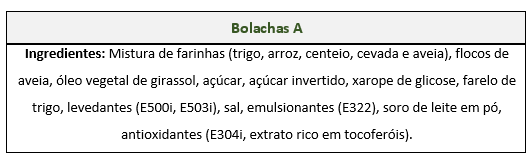


- 1. Dos seguintes conjuntos de ingredientes qual aquele que corresponde a açúcar?

| - Açúcar, açúcar invertido e antioxidantes - Açúcar e açúcar invertido - Açúcar, açúcar invertido e xarope de glicose - Açúcar - Açúcar, açúcar invertido e emulsionantes |  |
| --- | --- |

- 1. Analisando a lista de ingredientes, qual é o ingrediente que existe em maior quantidade nas bolachas?

| - Flocos de aveia - Açúcar - Açúcar Invertido - Farelo de Trigo - Emulsionantes - Soro de leite em pó | - Mistura de Farinhas - Óleo vegetal de Girassol - Xarope de Glicose - Levedantes - Sal - Antioxidantes |
| --- | --- |

**V. ATITUDES, COMPORTAMENTOS, COMPETÊNCIAS E PREFERÊNCIAS ALIMENTARES**

Para as seguintes frases, indique o seu grau de concordância com as mesmas (concordo plenamente, concordo em parte, nem concordo nem discordo, discordo em parte, discordo totalmente).

|  | Concordo Plenamente | Concordo em parte | Nem concordo nem discordo | Discordo em parte | Discordo totalmente | NÃO QUERO RESPONDER |
| --- | --- | --- | --- | --- | --- | --- |
| 1. Incentivo o meu filho a comer fruta. |  |  |  |  |  |  |
| 1. Em casa, o meu filho pode comer toda a fruta de que gosta. |  |  |  |  |  |  |
| 1. Frequentemente comemos fruta todos em família. |  |  |  |  |  |  |
| 1. A fruta não me sacia. |  |  |  |  |  |  |
| 1. Os vegetais não me saciam. |  |  |  |  |  |  |
| 1. Incentivo o meu filho a comer vegetais. |  |  |  |  |  |  |
| 1. Frequentemente comemos vegetais todos em família. |  |  |  |  |  |  |
| 1. Em casa, o meu filho pode comer todos os vegetais de que gosta. |  |  |  |  |  |  |
| 1. É importante para mim que o meu filho não consuma muito sal todos os dias. |  |  |  |  |  |  |
| 1. É saudável para o meu filho consumir menos sal. |  |  |  |  |  |  |
| 1. Comida sem sal não tem sabor. |  |  |  |  |  |  |
| 1. É importante para o meu filho não consuma muitas bebidas açucaradas. |  |  |  |  |  |  |
| 1. Eu gosto do sabor de bebidas açucaradas. |  |  |  |  |  |  |
| 1. É importante para mim evitar comprar alimentos com excesso de açúcar. |  |  |  |  |  |  |
| 1. É importante para mim evitar ter alimentos açucarados facilmente acessíveis para o meu filho. |  |  |  |  |  |  |
| 1. Eu consigo que o meu filho coma fruta à sobremesa. |  |  |  |  |  |  |
| 1. Eu sei preparar refeições com vegetais congelados. |  |  |  |  |  |  |
| 1. Eu consigo preparar refeições em que 1/3 do prato é ocupado por vegetais. |  |  |  |  |  |  |
| 1. Eu consigo preparar refeições com vegetais que o meu filho goste. |  |  |  |  |  |  |
| 1. Eu sei escolher os alimentos com baixo teor sal. |  |  |  |  |  |  |
| 1. Eu consigo preparar uma refeição saborosa sem adicionar sal. |  |  |  |  |  |  |
| 1. Eu sei escolher os alimentos com baixo teor de açúcar. |  |  |  |  |  |  |

**Insegurança alimentar**

**Sabemos que o preço dos alimentos pode ter influência nas escolhas que fazemos. As perguntas que se seguem têm a ver com a influência que as questões económicas podem ter nos hábitos alimentares. Para cada uma das seguintes perguntas, responda sim ou não.**

| **Questão** | **Não** | **Sim** | **Não quero responder** |
| --- | --- | --- | --- |
| 1. Nos últimos três meses, teve a preocupação de que a comida acabasse antes que tivesse dinheiro para comprar mais comida? |  |  |  |
| 1. Nos últimos três meses, os alimentos acabaram antes que tivesse dinheiro para comprar mais comida? |  |  |  |
| 1. Nos últimos três meses, o seu agregado familiar ficou sem dinheiro para ter uma alimentação saudável e variada? |  |  |  |
| 1. Nos últimos três meses, as pessoas do seu agregado familiar comeram apenas alguns poucos tipos de alimentos que ainda tinham porque o dinheiro acabou? |  |  |  |
| 1. Nos últimos três meses, algum adulto do seu agregado familiar deixou de fazer alguma refeição porque não havia dinheiro para comprar a comida? |  |  |  |
| 1. Nos últimos três meses, algum adulto do seu agregado familiar comeu menos do que achou que devia porque não havia dinheiro para comprar comida? |  |  |  |
| 1. Nos últimos três meses, algum adulto do seu agregado familiar sentiu fome, mas não comeu porque não tinha dinheiro para comprar comida? |  |  |  |
| 1. Nos últimos três meses, algum adulto do seu agregado familiar ficou um dia inteiro sem comer ou, teve apenas uma refeição ao dia porque não tinha dinheiro para comprar a comida? |  |  |  |
| 1. Nos últimos três meses, as crianças do seu agregado familiar não puderam ter uma alimentação saudável e variada porque não havia dinheiro para comparar comida? |  |  |  |
| 1. Nos últimos três meses, as crianças do seu agregado familiar comeram apenas alguns poucos tipos de alimentos que ainda havia neste domicílio porque o dinheiro acabou? |  |  |  |
| 1. Nos últimos três meses, alguma criança do seu agregado familiar comeu menos do que você achou que devia porque não havia dinheiro para comprar a comida? |  |  |  |
| 1. Nos últimos três meses, foi diminuída a quantidade de alimentos das refeições de alguma criança do seu agregado familiar porque não havia dinheiro suficiente para comprar a comida? |  |  |  |
| 1. Nos últimos três meses, alguma criança do seu agregado familiar deixou de fazer alguma refeição porque não havia dinheiro para comprar a comida? |  |  |  |
| 1. Nos últimos três meses, alguma criança do seu agregado familiar sentiu fome mas não comeu porque não havia dinheiro para comprar mais comida? |  |  |  |
